# Effect of Nano-SiO_2_/PVA Fiber on Sulfate Resistance of Cement Mortar Containing High-Volume Fly Ash

**DOI:** 10.3390/nano12030323

**Published:** 2022-01-19

**Authors:** Jingjing Huang, Zhongkun Wang, Dongsheng Li, Gengying Li

**Affiliations:** 1 Department of Civil Engineering, Shantou University, Shantou 515063, China; 15jjhuang2@stu.edu.cn (J.H.); lids@stu.edu.cn (D.L.); 2 College of Civil Engineering and Architecture, Wenzhou University, Wenzhou 325035, China; 13526578601@163.com; 3 College of Water Conservancy and Civil Engineering, South China Agricultural University, Guangzhou 510642, China

**Keywords:** sulfate resistance, nano-SiO_2_, PVA fibers, high-volume fly ash cement mortar, mechanical properties

## Abstract

Sulfate resistance of high-volume fly ash/cement mortars hybrid containing 0~1.5 wt.% of nano-silica (Nano-SiO_2_, NS) and 0~1.0 vol.% of polyvinyl alcohol (PVA) fibers was investigated in this study. Fly ash was replaced with Portland cement at levels of 60% by weight. The resistance to sulfate attack was investigated by exposing the mortars to 10 wt.% sodium sulfate (Na_2_SO_4_) solutions for 72 days, after which change in mass, compressive, and flexural strengths were determined. For comparison, the compressive and flexural strengths of cement mortar after 100 days of curing in water were also investigated. Microstructural deteriorations caused by sulfate attack were analyzed by using scanning electron microscope (SEM). The test results showed that the combination of NS and PVA fibers was effective in enhancing the mechanical properties and the resistance to sulfate attack. After 28 days curing, the hybrid addition of 1.5 wt.% NS and 1.0 vol.% PVA fibers increased the flexural strength by 90% over the control one without NS and PVA fiber. Moreover, regardless of PVA fibers content, due to the crystal nucleus and pore-filling effects, the adding of 0.5 wt.% NS increased the compressive strength by 67.1~118.2%. Chemical reaction took place between fly ash and Na_2_SO_4_ as no un-hydration particles could be observed in the samples immersed in Na_2_SO_4_ solutions for 72 days, while a lot of un-hydration fly ash particles could be found in the SEM image of mortar after 100 days curing in water. The chemical reaction production could increase the adhesive property and fill the pores of cement mortar. As a result, the compressive and flexural strengths of cement mortars after immersed in Na_2_SO_4_ solution for 72 days, were much higher than that after 28 days curing. Moreover, the compressive strength of mortars incorporating 1.0~1.5 wt.% NS was even higher than that after 100 days curing in water, indicating the combination of Nano-SiO_2_/PVA fiber is effective in enhancing the resistance to sulfate attack.

## 1. Introduction

Approximately 8% of the global CO_2_ emissions released into the atmosphere are due to Portland cement (PC) production. It is reported that the replacement of cement by using active admixtures in construction engineering could reduce about 1.5 billion tons of carbon dioxide emitted to the atmosphere from PC production [1]. In addition to being an environmentally friendly material, active admixtures attract attention due to their pozzolanic activity and good durability properties. Fly ash, one of the most frequently used active admixtures can replace part of cement as supplementary cementitious material. The replacement will reduce solid waste, natural resources for the production of cement, and the emission of harmful gases [2]. The replacement of cement with partly fly ash to produce cement mortar and concrete offers both environmental benefit and early flowing property [2,3,4,5]. Moreover, fly ash can reduce the heat of hydration of cement [5], and improve the long-term working performance of concrete [6,7,8,9,10,11]. As the government pays more attention to the greenhouse effect and environmental protection, high-volume fly ash cementitious materials (HVFA) will be widely studied as a more environmentally friendly and sustainable composite material [12]. However, fly ash has low activity, the replacement of cement with high-volume fly ash might greatly lower the strength of cementitious materials in the early stage. Some nano mineral particles can increase the activity of fly ash [13,14,15,16]. Nano-particles generally have a diameter of 1–100 nm resulting in a ultra-high surface energy and reactivity [17,18]. Nano-SiO_2_ (NS), one of the most commonly used nano mineral particles, can greatly enhance the activity of fly ash in the early stage of cement hydration [19]. Thus, this paper will investigate the effect of NS on the strength of HVFA.

HVFA is a brittle material with poor toughness. Cracks may occur under load and deformation, which will cause structural failure and damage, and seriously affect the durability and safety of cement concrete structures [20,21]. The reinforcing and toughening effect of fiber can effectively limit the generation and development of cracks [22]. Polyvinyl alcohol (PVA) fiber has the advantages of high elastic modulus and tensile strength, large deformation ability, acid and alkali corrosion resistance, good adhesion to cement, and environmental protection [23,24,25]. The incorporation of PVA fibers to HVFA cement materials can effectively enhance toughness, limit the development of cracks, and enhance the mechanical properties and durability of composite materials. This article will study the influence of nano-SiO_2_ and PVA fiber on the mechanics and sulfate resistance of HVFA cement mortar, and provide a theory for the practical engineering application of nano-SiO_2_/PVA fiber composite-modified HVFA cement concrete material.

Sulfate attack is one of the important problems threatening the durability of cementitious materials. Some of the sulfate sources are ground or surface waters and natural soils. Foundations and parts of structures in contact with groundwaters, soils or wastewater may be exposed to sulfate attack. Sulphate attack in concrete is primarily due to sulfate concentration in service environment but can also occur internally with delayed ettringite formation or aggregates with sulfide inclusions. Previous studies have shown that sulfate attack can cause the concrete to expand, crack, and pour, and thus loss of strength [26]. It is stated that in concrete produced with 100% PC, sulfate attack is significant due to the high C_3_A content of the PC. Better resistance to sulfate attack is expected for cementitious materials that contains low rate of PC. Although there are many studies on the resistance of fly ash concretes to sulfate attack, studies investigating the sulfate resistance of nano-SiO_2_/PVA fiber modified HVFA are very limited.

The motivation of this work is to determine the effect of nano-SiO_2_/PVA fiber addition on the properties of cement mortar containing high-volume of fly ash. The strength development up to 100 days and the resistance of sulfate attack by exposing samples to 10% sodium sulfate (Na_2_SO_4_) solutions for 72 days were tested. Microstructures of the composites were also analyzed by using scanning electron microscope (SEM). 

## 2. Materials and Methods

### 2.1. Materials

**Figure 1:**
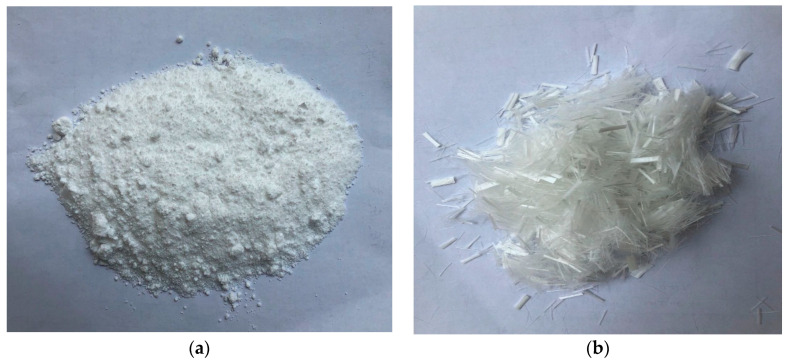
Appearance features of (**a**) NS and (**b**) PVA.

The P.O. 42.5R Portland cement was produced by Guangdong Tapai Cement Co., Ltd. (Guangzhou, China), and its physical and chemical properties are listed in Table 1. The fine aggregate is standard sand produced by Xiamen Aisiou Standard Sand Co., Ltd. (Xiamen, China), with a fineness modulus of 2.85 and density of 2.65 g/cm^3^. The properties of fly ash produced by the Shouxian Power Plant in China are shown in Table 1, and its main components are Al_2_O_3_ and SiO_2_. Fly ash used in this paper belong to low-calcium fly ash as its CaO content is less than 10%. NS (Figure 1a) is produced by Hangzhou Wanjing New Material Co., Ltd. (Hangzhou, China), and its diameter is about 30~50 nm. The main component of NS is SiO_2_ with low crystallinity. The superplasticizer is a brown-yellow powder, and the water reducing efficiency is 25%. PVA fiber is K-II type fiber produced by Japan Co., Ltd., (Tokyo, Japan) and its characteristics are shown in Figure 1b and Table 2. 

### 2.2. Specimen Preparation and Test Methods

Two series of cement mortar respectively containing 60 wt.% or 80 wt.% of fly ash were produced in this paper, the mix proportions of the unfired bricks are given in Table 3, where the volume content of PVA fiber was 0, 0.2%, 0.5%, and 1.0% and NS mass content was 0, 0.5%, 1.0%, and 1.5%, respectively. 

The specimens were prepared using the following steps:

(1) Weighting cement, sand, water, superplasticizers, FA, NS and PVA fibers;
(2) Dry mixing cement, sand, FA, NS and superplasticizers for 2 min;
(3) Adding water into the above concrete mixture and mixing for another 2 min;
(4) Adding the PVA fiber to the mixture while stirring and mixing for another 3 min;
(5) Pouring the mix into oiled molds, and smoothening the surfaces of specimens and then covering them with wet clothes;
(6) Demolding the specimens after 24 h curing and then curing them in air at 25 ± 2 °C until 28-day age before testing (water specimens every day for the first 7 days).

Six 100 mm × 100 mm × 100 mm cubic specimens were prepared for compressive strength tests, and three prismatic specimens of 160 mm (length) × 40 mm (width) × 40 mm (height) in dimensions were prepared for flexural strength tests with a supporting span of 100 mm according to the “Test Method for Strength of Cement Mortar Sand” of Chinese Standard Specification (GB/T 17671-1999) [27], and they were compacted by vibration. An automatic compression testing machine CMT-5105 was used in displacement control mode to conduct the three-point bending test with a loading rate of 0.05 mm/min. A servo-hydraulic YAW-4306 testing system was utilized to perform the compressive test with a loading rate of 0.5 MPa/s. The samples were first cured in water for 3 days and then in air at room temperature (24 ± 5 °C) to different testing ages of 28 and 100 days.

**Figure 2:**
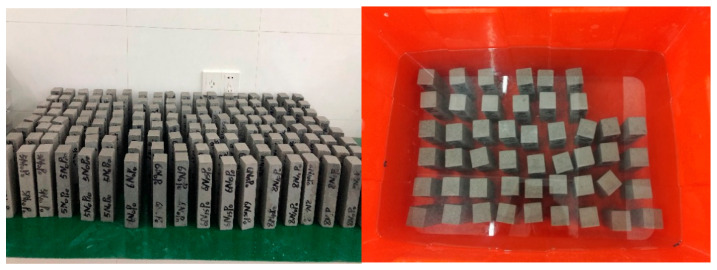
Samples for sulfate corrosion resistance test.

Sulfate resistance was conducted in plastic pot in accordance with “Standard for long-term performance and durability test methods of ordinary concrete” (GB/T 50082-2009) [28] and “Test methods for cement sulfate corrosion resistance” (GB/T 749-2008) [29]. Specimens after 28-day curing (first cured in water for 3 days and then in air at room temperature (24 ± 5 °C)) were subjected to sulfate attack as shown in Figure 2. Specimens (40 mm × 40 mm × 160 mm) were divided into two groups. One group of specimens was cured under the standard curing room, and the other group was immersed in 10% sodium sulfate solution. The mass of specimens in sodium sulfate solution was tested every 10 days, and the flexural and compressive strengths were test at the age of 100 days. The degree of deterioration was assessed by measuring the changes in mass and compressive strength after 72 days of exposure to sulfate.

Morphology and microstructure studies were also performed with a Quanta 200 field emission environmental scanning electron microscope (SEM). Three samples with a size about 1 cm × 1 cm × 1 cm were prepared for each mix at the age of 28 days from failed specimens after the strength test. These samples were kept in alcohol till inspecting in the SEM, and were gold-coated before examination.

## 3. Results and Discussion

### 3.1. Compressive and Flexural Strength before Immersion in Sulfate Solution

**Figure 3:**
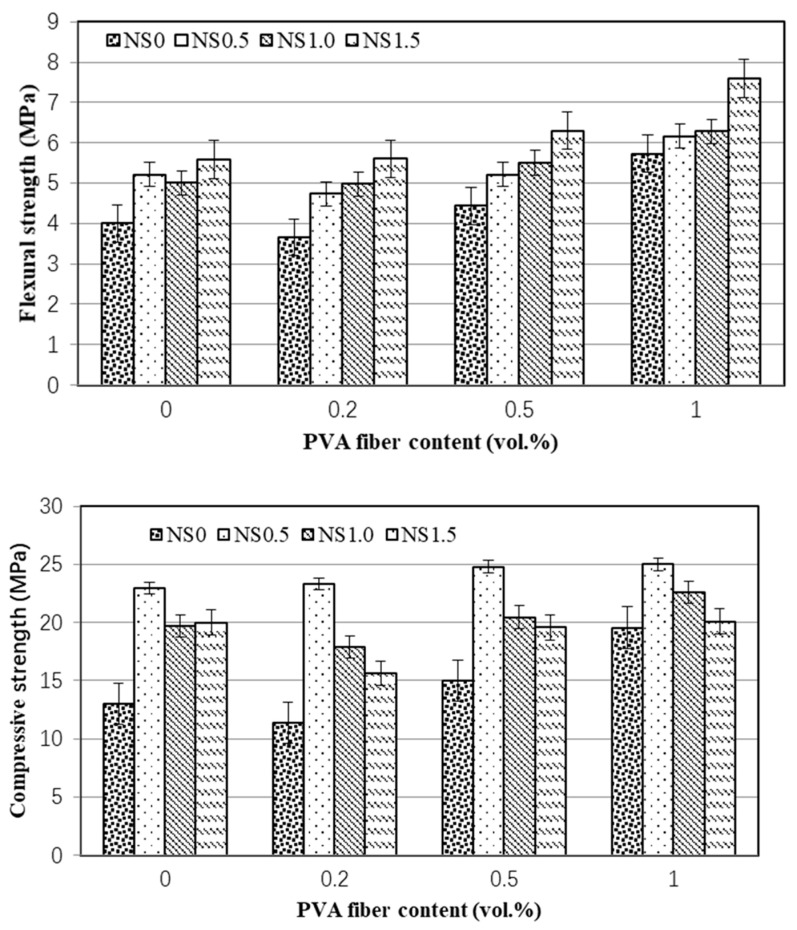
Effect of PVA fiber content on 28-days flexural and compressive strength of mortar.

**Figure 4:**
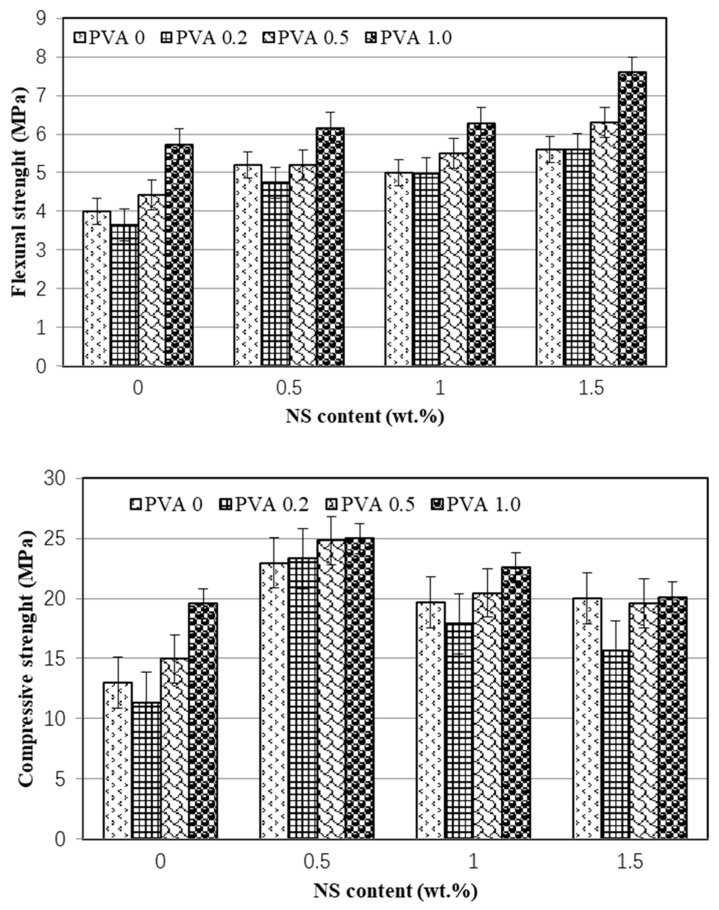
Effect of NS content on 28-days flexural and compressive strength of mortar.

The 28-day mechanical properties of all mixes are presented in Table 4 and Figure 3 and Figure 4. Clearly, the combined adding of NS and PVA fibers influences the flexural and compressive strength of cement mortar with high-volume fly ash greatly. As shown in Figure 3, regardless of the replacement mass of fly ash, the flexural strength of cement mortar increased with the increase of PVA fiber content, and the adding of 1.0 vol.% PVA fiber increased the 28-days flexural strength of cement mortar by 18.3~43.2%. As for the 28-days compressive strength, the enhancing effect of PVA fiber varied with the addition of NS, and the higher the NS content, the higher the enhancement effect of PVA fiber will be. As shown in Figure 3, the incorporating of 0.2 vol.% PVA fiber decreased the compressive strength of mortar by −12.7%, while combined with 1.5 wt.% NS, the compressive strength increased by 20.1%. Moreover, Figure 3 shows that the adding of PVA fiber has a negative effect on the compressive strength, which is possibly caused by the poor dispersiveness of PVA fiber within cement mortar. The soft PVA fiber agglomerated particles may cause the stress concentration, leading to reduction of the ultimate bearing capacity of cement mortars.

With regard to NS, as shown in Figure 4, the 28-days flexural strengths increased with the increase of NS. The addition of 0.5%, 1.0%, and 1.5% NS increases the 28-day flexural strength by 30.0%, 25.0%, and 39.8%, respectively. The combination with PVA fibers may further increase the flexural strength, and the optimal proportion of mortar was the hybrid addition of 1.5 wt.% NS and 1.0 vol.% PVA fibers, which had the highest flexural strength of 7.6 MPa, about 90% higher than that of the control one (NS0-PVA0) without NS and PVA fiber. Moreover, Figure 4 shows, when taking 28-days compressive strength, the optimal content of NS was about 0.5 wt.% regardless the PVA fibers content. When compared to the mortars only containing PVA fibers, the adding of 0.5 wt.% NS increased the compressive strength by 67.1~118.2%, which may be caused by the crystal nucleus and pore-filling effects of NS as pointed out by authors [30,31].

**Figure 5:**
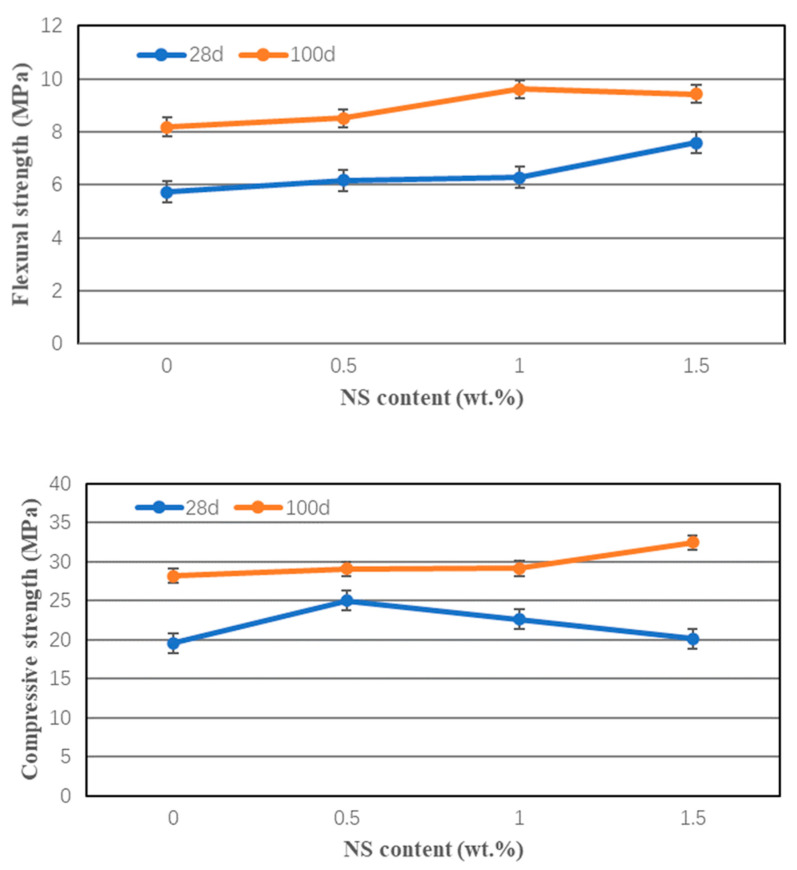
The flexural and compressive strength of HVFA mortar at 28 and 100-days.

This paper also investigated the curing age on the combined effect of NS and 1.0 vol.% PVA fibers on the flexural and compressive strength of cement mortar, and the results are shown in Figure 5. Clearly, the curing age influences the flexural and compressive strength greatly, and the 100-days strength was significantly higher than that of the 28-days strength as expected. For example, the 100 days compressive and flexural strengths of cement mortar combination of 1.0 wt.% NS and 1.0 vol.% PVA fiber were about 29% and 53% higher than that after 28 days of curing. Moreover, this figure shows that the flexural strength after 28 and 100 days curing generally increased with the increase of NS content. Similar tendency was observed for the 100-days compressive strength, and the incorporation of 1.5 wt.% NS and 1.0 vol.% PVA fibers had the highest compressive strength of about 33 MPa.

### 3.2. Morphology and Microstructure 

**Figure 6:**
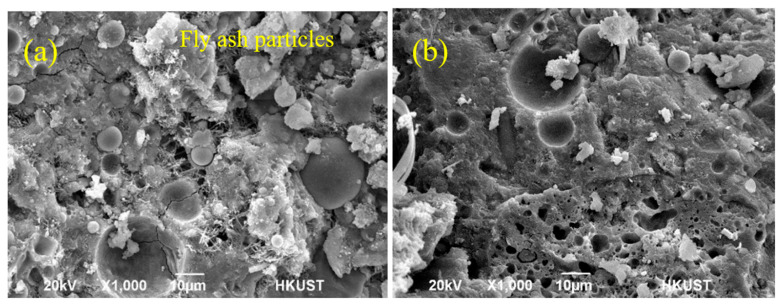
SEM images of NS1.5-PVA1.0 ((**a**). after 28 days curing, (**b**). after 100 days curing).

**Figure 7:**
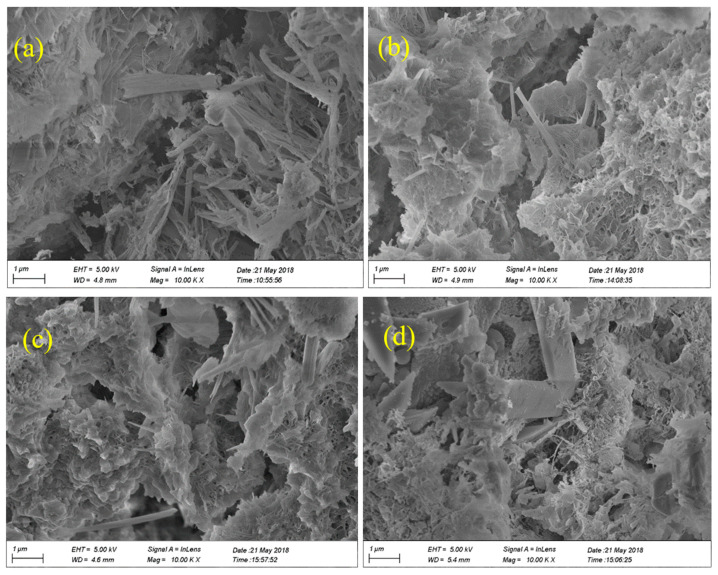
SEM images of cement mortar with different NS content immersed in 10% sodium sulfate solution for 72 days ((**a**) NS0-PVA0, (**b**) NS0.5-PVA0, (**c**) NS1.0-PVA0, (**d**) NS1.5-PVA0).

Microstructure plays an important role in determining the mechanical properties and durability properties of concrete. To better understand the effect of NS and PVA on the properties of cement mortar with high-volume fly ash, the mineralogy and microstructure of NS1.5-PVA1.0 incorporating 1.5 wt.% NS and 1.0 vol.% PVA fiber were examined by SEM after 28 and 100 days of curing. Moreover, the microstructure of mortars after immersing in 10% sodium sulfate solution for 72 days was also investigated. The results are shown in Figure 6 and Figure 7 for comparison and analysis.

Figure 6a,b shows the SEM images of NS1.5-PVA1.0 after 28 and 100 days of curing. Obviously, there is a significant amount of spherical unreacted fly ash particles with smooth surface in Figure 6a. Moreover, some big pores, cracks, and needle crystals can be found in this figure, indicating low strength at the age of 28 days. In Figure 6b, spherical unreacted fly ash particles with smooth surface could also be observed for NS1.5-PVA1.0 after 100 days of curing. However, from the comparison of Figure 6a to Figure 6b, it can be found that the amount of unreacted fly ash particles in the 100-days sample was much lower than that of the 28-days sample; moreover, the content of big pores and cracks as well as needle crystals of mortar after 100 days of curing was also lower than that after 28 days of curing. The denser microstructure of mortar after 100 days curing led to a higher mechanical property as shown in Figure 5, in which the 100 days compressive and flexural strengths of NS1.5-PVA1.0 were respectively about 64.0% and 24.1% higher than that after 28 days of curing. The improvement mechanism is due to the hydration of fly ash increase with the increase of curing age.

Figure 7a–d shows the SEM images of cement mortars incorporating 0, 0.5, 1.0, and 1.5 wt.% NS (NS0-PVA0, NS0.5-PVA0, NS1.0-PVA0, and NS1.5-PVA0) after immersing in 10% sodium sulfate solution for 72 days. No fly ash particles can be found in these figures, indicating the chemical reactions possibly take place between fly ash and sodium sulfate solution. The chemical reactions may include the following three steps:

(1) Secondary hydration between the active Al_2_O_3_ of fly ash and the hydration of Ca(OH)_2_ cement
  Al_2_O_3_ + aH_2_O + bCa(OH)_2_ → bCaO Al_2_O_3_ nH_2_O (1)
(2) Chemical reaction between hydrated calcium aluminate and sodium sulfate solution
  bCaO·Al_2_O_3_·nH_2_O + nH_2_O + xNa_2_SO_4_ → bCaO·Al_2_O_3_·xNa_2_SO_4_·mH_2_O (2)
(3) Chemical reaction between the active Al_2_O_3_ of fly ash and sodium sulfate solution
  Al_2_O_3_ + nH_2_O + Na_2_SO_4_ → Na_2_SO·Al_2_O_3_·nH_2_O (3)

The above chemical reaction not only dissolves the glassy coated on fly ash leading to the collapse and decomposition of fly ash but also increases the adhesive property of cementitious binder. 

In addition, Figure 7 shows there exists a lot of needle crystals in cement mortars, which is a further proof of the chemical reaction between fly ash and sodium sulfate solution. Moreover, Figure 7 shows that the content of NS influences the microstructure; the more the NS content, the denser the microstructure will be, indicating NS is effective in improving the properties of cement mortar.

### 3.3. Compressive and Flexural Strength after Immersion in Sulfate Solution

**Figure 8:**
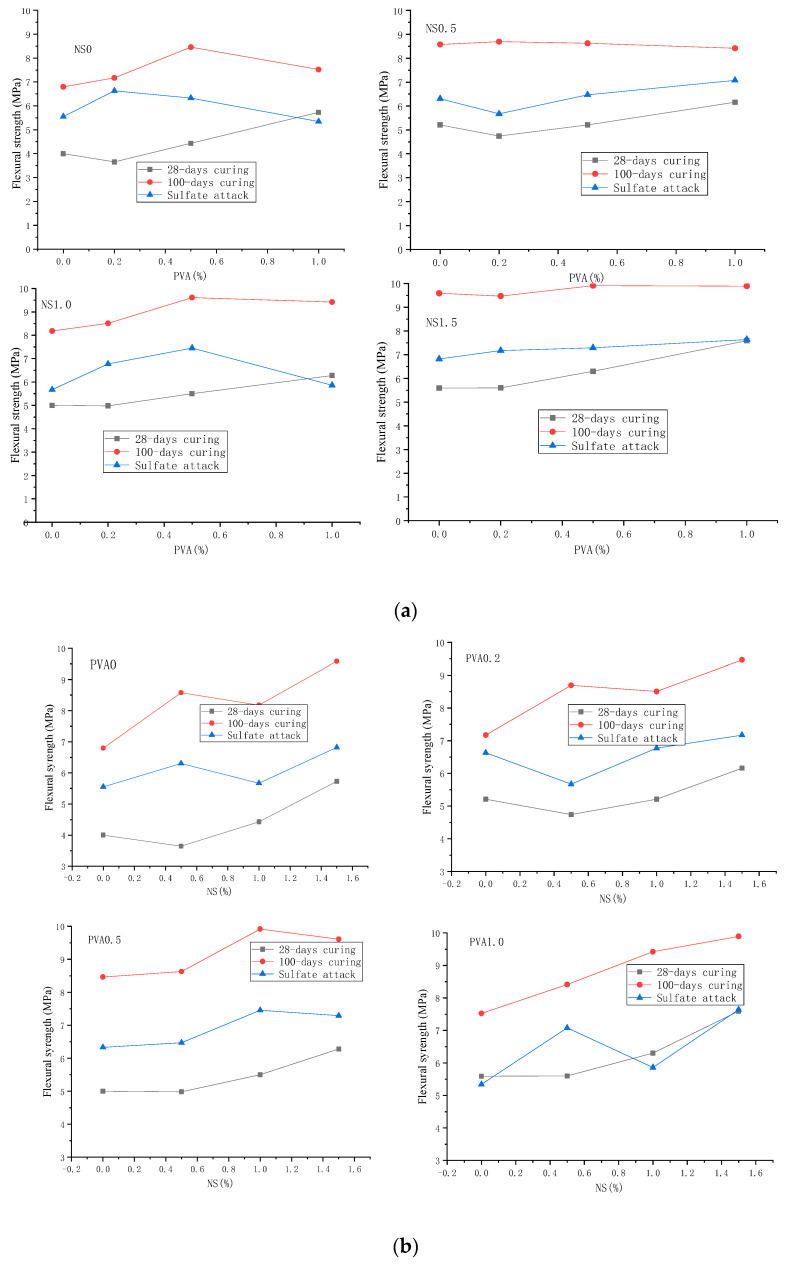
Flexural strength of mortar with 60% FA content after sulfate erosion. (**a**) Effect of PVA fibers content. (**b**) Effect of NS content.

**Figure 9:**
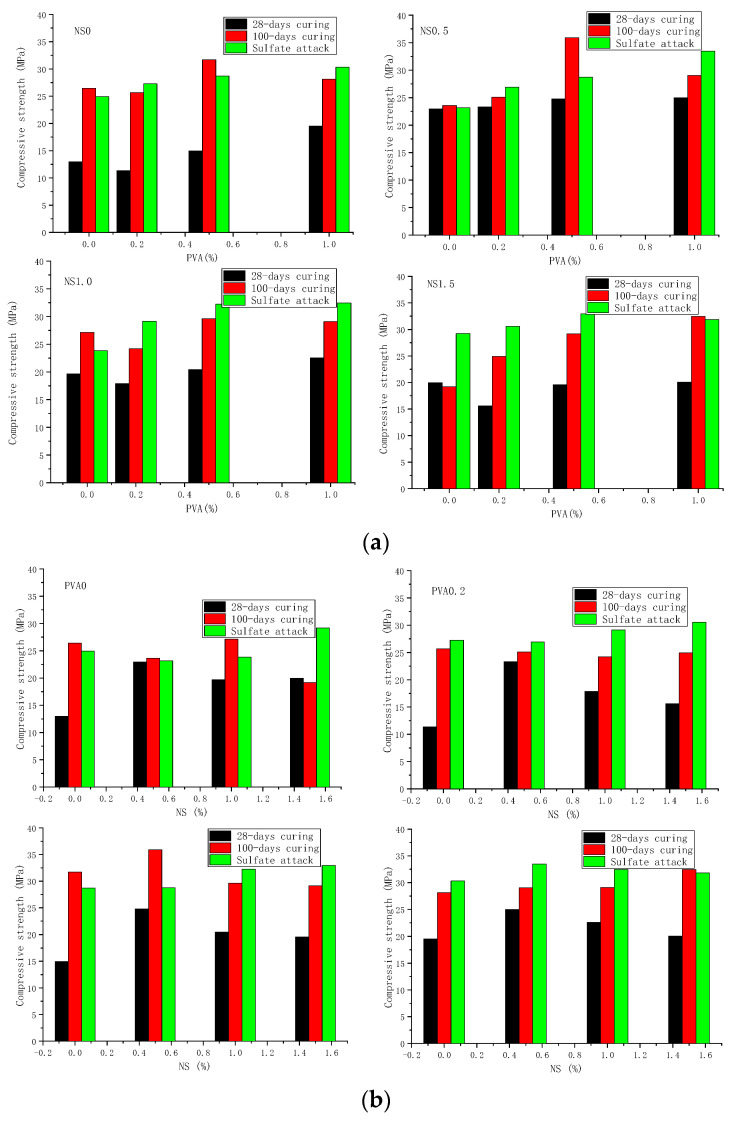
Compressive strength of mortar with 60% FA content after sulfate attack. (**a**) Effect of PVA fibers content. (**b**) Effect of NS content.

The residual flexural and compressive strengths of mortar specimens containing 60% FA after immersing in 10% sodium sulfate solution for 72 days are presented in Figure 8 and Figure 9. From these figures, it can be found that the samples have good sulfate resistance as the residual flexural strengths after 72 days sulfate attack for all samples were generally higher than that of 28-days flexural strengths. As shown in Figure 8, the mortar incorporating 1.5 wt.% NS and 1.0 vol.% PVA fiber had the highest residual flexural strength of 7.64 MPa, which was about 91% higher than the 28-days flexural strength of control samples (4.0 MPa). However, when comparing the residual flexural strength with that after 100 days of water curing (100-days flexural strength), as shown in Figure 8, the residual flexural strength of all specimens is much lower than the 100-days flexural strength. This low flexural strength may be caused by the destruction of PVA fibers on the sodium sulfate solution which need further investigation to gain insight into the mechanism.

Figure 9 shows the effect of sulfate attack on the compressive strength of cement mortar hybrid incorporating NS and PVA fibers. After 72 days sulfate attack, the compressive strengths were generally higher than that cured in air for 28 days. The main reason is that the chemical reaction products according to Equations (1)–(3) could not only enhance the bonding force but also fill the pores of mortar as shown in Figure 7, leading to the enhancement in compressive strength. Moreover, these figures show that the mortar incorporating 1.5 wt.% NS and 0.5 vol.% PVA fiber had the highest residual compressive strength of 32.97 MPa, which was about 1.54 times higher than the 28-days compressive strength of control samples (13.0 MPa). Moreover, comparing the residual compressive strength with that after 100 days of water curing (100-days compressive strength), as shown in Figure 9, the residual compressive strength of mortars containing 1.0 and 1.5 wt.% NS was higher than the 100-days compressive strength, indicating the enhancement effect of NS on the sulfate resistance of cement mortar containing high-volume fly ash.

The sulfate resistance coefficient of HVFA mortar is defined as the ratio of the strength after sulfate erosion to the strength of the specimen under normal curing. When the addition of NS is 0.5%, and the PVA fiber is 0.2%, 0.5%, and 1.0%, the corrosion resistance coefficients of flexural strength are 0.78, 0.75, and 0.85, while the corrosion resistance coefficients of compressive strength are 1.05, 0.89, and 0.93, respectively. The flexural corrosion resistance coefficients of all HVFA mortars are lower than 1, while the compressive corrosion resistance coefficients are basically greater than 1.

After sulfate attack, the strength of mortar is not decreased obviously, and some group of mortar increased when it is compared to the normal curing mortar. The reason is that the products of sulfate erosion can fill the holes in the mortar, which can improve the mortar compactness (as showed in Figure 7). The micro-structure of unmixed mortar is shown in Figure 7a. There are many holes, and big cracks can be seen in the figure. Even the sulfate erosion products fill some holes. There are almost no holes and cracks in the 0.5% NS-modified mortar (Figure 7b). When the addition of NS is 1.0% (Figure 7c) and 1.5% (Figure 7d), there are still some cracks and holes in the mortar. However, the width of crack in the 1.0% and 1.5% NS modified mortar is much smaller than the mortar without NS, and also, the number of holes is less than control mortar.

### 3.4. Mass Change

**Figure 10:**
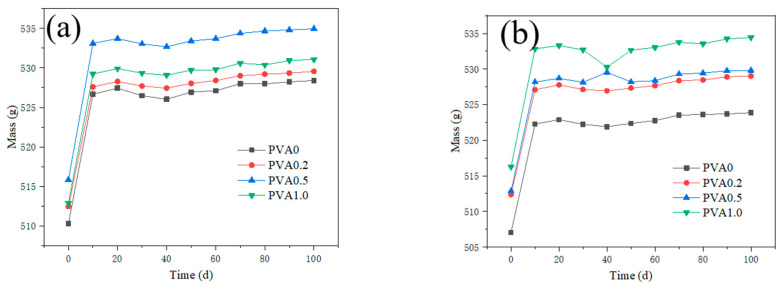
Effect of PVA content on mass of mortar after sulfate attack. ((**a**) NS0.5, (**b**) NS1.5).

**Figure 11:**
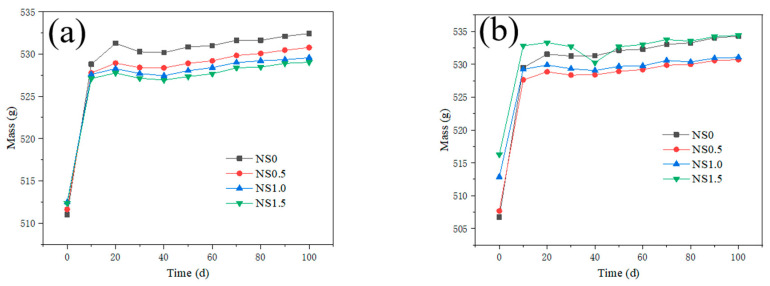
Effect of NS content on mass of mortar after sulfate attack. ((**a**) PVA0.2, (**b**) PVA1.0).

The mass of mortar after sulfate attack is presented in Figure 10 and Figure 11. When the NS content is 1.0% and 1.5%, the mortar mass of different content PVA fiber is showed in Figure 10a,b. With the increase of sulfate attack time, the mass of the specimens increases rapidly at first, then increases slowly. Comparing the mass of the specimens with different amounts of PVA after sulfate erosion, it is found that with the increase in addition of the PVA fiber, the growth rate of the specimen mass basically increases. The incorporation of PVA fiber may bring bubbles into mortar. The more air bubbles are introduced into the mortar, the more sulfate corrosion products will penetrate into the interior of mortar during the sulfate erosion process, and the higher the mass growth rate will be.

When the PVA content is 0.2% and 1.0%, the mortar masses of different contents of NS are shown in Figure 11a,b. It can be seen from Figure 11a that with the increasing addition of the NS, the mass of the specimens decreases. The mass decrease indicates that NS, as a fine particle, has the effect of crystal nucleation, and improves the hydration rate of the cementitious material [19,32]. In addition, NS can effectively fill the internal pores of the cement mortar, making the mortar structure denser and not easily sulfate corroded, and improving its durability. However, when the addition of PVA fiber is 1.0%, the mortar mass decreases first and then increases. Because the addition of PVA fiber is too large, with more bubbles in the mortar, the effect of NS is not obvious.

### 3.5. Discussion

It can be seen from the above experimental results that NS can effectively enhance the flexural and compressive strength of the modified mortar. This is due to two reasons [33,34]: (1) NS is a very fine particle with a large specific surface area and super high surface energy and reactivity. Moreover, it can react with cement hydration products to improve the hydration degree of cement and FA. (2) As fine particles, NS can fill the internal pores of cement paste well, and it can improve the compactness of the cement matrix, reducing the possibility of the generation and development of micro-cracks, and effectively improving the mechanical properties.

PVA fiber significantly improves the flexural strength of the modified mortar, but the increase in compressive strength is small. With the addition of PVA content, the flexural strength of mortar increases. PVA fiber has a good compatibility with cement, and it is dispersed in cement paste, like many short steel bars, which limits the generation of cracks and effectively inhibits the expansion of micro-cracks to macro-cracks. Besides, PVA fiber has a very good bridging effect. When cracks occur, the fibers penetrate the cracks and distribute the stress to the surrounding cement paste, which can reduce stress concentration, limit the extension and development of cracks, and improve the mechanics of the modified cement mortar performance [35]. However, PVA fibers may introduce gas into the mortar [36], resulting in no significant increase in the compressive strength of the mortar.

The composite addition of NS and PVA fiber improves the mechanical properties and sulfate resistance of cement mortar. The main reason is that NS (as a nanometer particle) can effectively fill pores, and it has a crystal nucleation effect. Moreover, it can promote the hydration of cement and FA, producing more hydration products and compacting structure. PVA fibers can effectively restrict the expansion of cracks, but may introduce gas into the mortar during the stirring process. Therefore, the composite addition of NS and PVA fiber can fill the pores of the cement paste, improving the compactness of the structure and effectively preventing the sulfate erosion process. Besides, when the sulfate corrosion products swell inside the structure to generate stress, PVA fiber can play a role in preventing cracks, limit the development of cracks, and improve the sulfate resistance of the structure.

## 4. Conclusions

1. The single addition of PVA fiber can significantly improve the flexural strength of the high-volume fly ash mortar, and the higher the addition of PVA fiber the higher the strength. Compared to the control group, the addition of 1.0% PVA can increase the flexural strength by 43%.
2. Compared to the control mortar, the single addition of NS can increase the flexural and compressive strength of the mortar. When the NS addition alone is 1.5%, the flexural and compressive strength increased by 40% and 54%, respectively. Moreover, the single addition of NS can effectively reduce the mass growth rate and the sulfate expansion products after sulfate attack.
3. The composite addition of NS and PVA fiber can significantly enhance the flexural and compressive strength of the modified mortar. When the addition of PVA is 1.0% and the NS is 1.0% and 1.5%, the flexural strength of the modified mortar is increased by 57% and 90%, and the compressive strength is increased by 74% and 55%.
4. The resistance to sulfate attack of the mortar modified by NS and PVA has been greatly improved. Combining 1.5% NS and 1.0% PVA fibers showed optimum property, in which the mass loss after immersing in 10% sodium sulfate solution for 72 days was about 16% lower than that of the control one, while the compressive and flexural strength increased by 23% and 39%, respectively. The SEM test result indicates that adding hybrid NS and PVA fibers is effective in limiting the generation of sulfate erosion products and keeping materials from expansive failure after sulfate attack.

## Figures and Tables

**Table 1:** Physical and chemical properties of binder.

	SiO_2_	Al_2_O_3_	CaO	MgO	Fe_2_O_3_	K_2_O	MnO	SO_4_	LOI	Specific Surface, Blaine, m^2^ kg^−1^	Compressive Strength, 28-Day, MPa
Cement	18.3	4.5	62.4	2.1	0.3	1.5	2.3	2.97	0.34	350	50.6
Fly ash	46.69	33.76	8.46	-	6.05	1.24	-	1.34	2.1	389	-
SiO_2_	99.5	-	-	-	-	-	-	-	-	2133	-

**Table 2:** The main properties of PVA.

Diameter/mm	Length/mm	Density/g·cm^−3^	Tensile Strength/MPa	Elastic Modulus/GPa	Elongation/%	Aspect Ratio
0.038	12	1.3	1092	30	7	316

**Table 3:** Mix ratio of cement mortars with high-volume of fly ash.

Material	Mix Proportions				Nano-SiO_2_ (w)/%	PVA Fiber (vol)/%	Superplasticizer (w)/%
	Water	Cement	Fly Ash	Sand			
NS0-PVA0	220	220	330	1100	0	0	1
NS0-PVA0.2	220	220	330	1100	0	0.2	1
NS0-PVA0.5	220	220	330	1100	0	0.5	1
NS0-PVA1.0	220	220	330	1100	0	1	1
NS0.5-PVA0	220	220	330	1100	0.5	0	1
NS0.5-PVA0.2	220	220	330	1100	0.5	0.2	1
NS0.5-PVA0.5	220	220	330	1100	0.5	0.5	1
NS0.5-PVA1.0	220	220	330	1100	0.5	1	1
NS01.0-PVA0	220	220	330	1100	1	0	1
NS01.0-PVA0.2	220	220	330	1100	1	0.2	1
NS01.0-PVA0.5	220	220	330	1100	1	0.5	1
NS01.0-PVA1.0	220	220	330	1100	1	1	1
NS01.5-PVA0	220	220	330	1100	1.5	0	1
NS01.5-PVA0.2	220	220	330	1100	1.5	0.2	1
NS01.5-PVA0.5	220	220	330	1100	1.5	0.5	1
NS01.5-PVA1.0	220	220	330	1100	1.5	1	1

**Table 4:** Flexural and compressive strength of the mortar with different mix ratio.

Coad Number	60% FA	
	Flexural Strength (MPa)	Compressive Strength (Mpa)
NS0-PVA0	4.00	13.00
NS0-PVA0.2	3.65	11.35
NS0-PVA0.5	4.43	14.98
NS0-PVA1.0	5.73	19.56
NS0.5-PVA0	5.21	22.95
NS0.5-PVA0.2	4.74	23.33
NS0.5-PVA0.5	5.21	24.81
NS0.5-PVA1.0	6.16	25.00
NS1.0-PVA0	5.00	19.68
NS1.0-PVA0.2	4.98	17.90
NS1.0-PVA0.5	5.50	20.46
NS1.0-PVA1.0	6.28	22.58
NS1.5-PVA0	5.59	20.00
NS1.5-PVA0.2	5.60	15.63
NS1.5-PVA0.5	6.30	19.59
NS1.5-PVA1.0	7.59	20.10

## Data Availability

The data used to support the findings of this study are available from the corresponding author upon request.
